# Two decades of agricultural drought impacts: remote sensing insights into vegetation productivity and phenological change in semi-arid Botswana

**DOI:** 10.1007/s10661-026-14996-w

**Published:** 2026-01-30

**Authors:** Felicia O. Akinyemi, Valerie Graw

**Affiliations:** 1 Geomatics, Department of Environmental and Life Sciences, Karlstad University, Universitetsgatan 2, 651 88 Karlstad, Sweden; 2 Land Systems and Sustainable Land Management, Institute of Geography, University of Bern, Hallerstrasse 12, 3012 Bern, Switzerland; 3 Geomatic Research Group (GRG), Ruhr-University Bochum (RUB), Universitaetsstrasse 150, 44801 Bochum, Germany

**Keywords:** Weighted vegetation condition index, Drought, Phenology, Time series analysis

## Abstract

Vegetation phenology needs to be considered in the assessment of agricultural drought severity, especially in drylands such as Botswana. Using Remote Sensing time-series data (2000 – 2020), this study evaluated vegetation productivity using the annual sum of the Enhanced Vegetation Index (EVI_sum_) and computed changes as trends (EVIsum_trend_) in agricultural lands comprising grasslands and croplands. To assess agricultural drought severity, a weighted linear combination was applied to the EVI-based Vegetation Condition Index (VCI_wlc_) and compared to the conventional Standardized Precipitation Index. To detect how agricultural drought has impacted vegetation phenology, we identified dynamics in vegetation greenup, maturity, peak, senescence and dormancy and correlated these to the EVI_sum_ and VCI_wlc_. Differentiating between croplands and grasslands, vegetation productivity in grasslands was consistently lower than in croplands during droughts. The seasonal agriculture-related phenology, such as late vegetation greenup — the start of the season — is correlated with reduced vegetation productivity and severe agricultural droughts as evidenced by lower EVI_sum_ and VCI_wlc_ values, respectively. Notable phenology patterns include delayed greenup in grasslands compared to croplands, while the peak period often overlaps between grasslands and croplands. Overall, phenological shifts detected over agricultural lands in Botswana were about 58 days delayed greenup between the earliest and latest detection, whereas for peak, senescence and dormancy, delays were between 60 and 61 days. Findings provide valuable insights into how vegetation responds over time to changing environmental stressors such as drought. Understanding and monitoring how drought impacts agriculture-related phenology in dryland ecosystems is essential to informing national drought policies.

**Supplementary Information:**

The online version contains supplementary material available at 10.1007/s10661-026-14996-w.

## Introduction

Knowledge about drought impacts on coupled social and ecological systems such as agriculture is required to adapt to climate change. Across scales, these systems are increasingly under stress (Bukhari et al., 2019; Cohen et al., 2021; Smith et al., 2009; Webb et al., 2017) as agriculture is affected by climatic changes (Fernández et al., 2019; Hatfield et al., 2011; Solh & van Ginkel, 2014). This is particularly true in drylands where a deficit in rainfall during droughts further constrains vegetation growth and productivity (Ghazaryan et al., 2020; Vicente-Serrano et al., 2024). It is well known that crops are sensitive to drought-induced changes in moisture conditions during the different stages of growth (Das & Pandey, 2025; Hassan et al., 2024; Jones & Thornton, 2003; Kuri et al., 2019). Moreover, agricultural productivity is being reshuffled across regions by climate and other factors (Bortz & Toftum, 2023; Khadka et al., 2025; Porfirio et al., 2018; Reynolds et al., 2018; Torquebiau et al., 2016). Hence, studies conducting drought assessment in agricultural contexts increasingly rely on Remote Sensing as complementary to place-based methods (Akinyemi, 2021) since it provides timely and spatially explicit data for assessing plant growth and managing agricultural outcomes. Widely used are satellite image time-series of rainfall and vegetation indices for drought and phenology assessment (Chivasa et al., 2017; Schreier et al., 2020).

Remote Sensing-based studies considering the spatiotemporal variations in phenology are often not focused on agricultural phenology (e.g., Ivits et al., 2014; Adole et al., 2018; Sun et al., 2023). Increasingly, agriculture-related phenology in the context of drought is of interest (e.g., Cohen et al., 2021; Wang et al., 2016). In Southern Africa, where intra- and interannual rainfall variability is high and incessant droughts impact agricultural productivity (Chipanshi et al., 2003; Mangani et al., 2019), phenology studies are few (e.g., Graw et al., 2020; Kombani et al., 2025). Graw et al. (2020), in their assessment of the Eastern Cape province, South Africa, found that drought impacts on crops depended mainly on the growth stage when the drought occurred. Thus, knowledge is to be gained from integrating the analysis of drought timing during the phenological growing cycle into assessing drought impacts.

Examining the case of Botswana, this study assessed changes to vegetation productivity in response to agricultural drought in agricultural lands, comprising grasslands and croplands. Drought reduces the amount of soil water available to plants, affects crop yield and livestock due to limited fodder availability (Lu et al., 2019). As Botswana is a dryland, drought is a recurrent phenomenon with an average return period of about two years. This makes timely vegetation monitoring and drought assessment critical for food security and drought management (Government of Botswana — GoB, 2019). For operational purposes in Botswana, drought is understood as a deficiency in rainfall regarding the timing, spatio-temporal distribution, rainfall amount and the severity of the deficiency to negatively affect plants, wildlife, water availability and food security (Manthe-Tsuaneng, 2014). As Botswana’s agriculture is predominantly rainfed and highly vulnerable to drought, its farming systems are reminiscent of African rainfed agriculture. Studies have found changes to the seasonality and intensity of rainfall, including an increasing number of dry spells and droughts (Adelabu et al., 2011; Byakatonda et al., 2018a; Maruatona & Moses, 2021). For example, Adelabu et al. (2011) found that the onset of rain shifted by at least 40 days during the 2008–2009 cropping season in Barolong, Southern Botswana with reference to the baseline 1960–1961 onset dates. A more recent study assessing phenology trends, which is not related to agriculture, further confirms such shifts in Botswana (Kombani et al., 2025). They found that the start of the season, ranging from 269 to 365 day of the year, extended by about 4 days decade^−1^, whereas the end of season by 0.8 days decade^−1^ with a shortening of the length of season by 1.5 days decade^−1^. Studies have equally found likely future changes in rainfall onset, cessation, and duration as well as increases in drought frequency and severity at varying global warming levels over Botswana (e.g., Akinyemi & Abiodun, 2019; Nkemelang et al., 2018; World Bank, 2021). Such shifts in the timing and magnitude of phenology and changes in the patterns of climate variables have implications for the resilience of agricultural systems. With more than 70% of Botswana’s rural population dependent on agriculture or associated agricultural activities, livelihoods are largely climate-impacted (Ministry of Finance and Development Planning — MFDP, 2010; Statistics Botswana, 2015a).

This study uses remote sensing-based time-series data from 2000 to 2020 to examine agricultural drought severity and impacts on vegetation phenology in Botswana. The two-decade study period was chosen to coincide with periods of drought with varying magnitudes since the beginning of the twenty-first century as identified in several studies (e.g., Akinyemi, 2021; Toreti et al., 2024; World Bank, 2021). With a primary focus on agriculture-related phenology, we considered five indicators (i.e., vegetation greening, maturity, peak, senescence and dormancy) in persistent croplands and, for comparison, in grasslands. Objectives are to i) conduct a country-wide spatio-temporal assessment of vegetation productivity and change, ii) detect drought years using the weighted linear combination (WLC) of the Vegetation Condition Index in comparison with the conventional Standardized Precipitation Index (SPI), and iii) examine agricultural drought interactions with seasonal dynamics of the phenology indicator. Using Remote Sensing-based time-series data, the main motivation for this study is the need to assess agricultural drought impacts on agriculture-related vegetation productivity and phenology to address the risk of drought.

## Background to study area

**Fig. 1:**
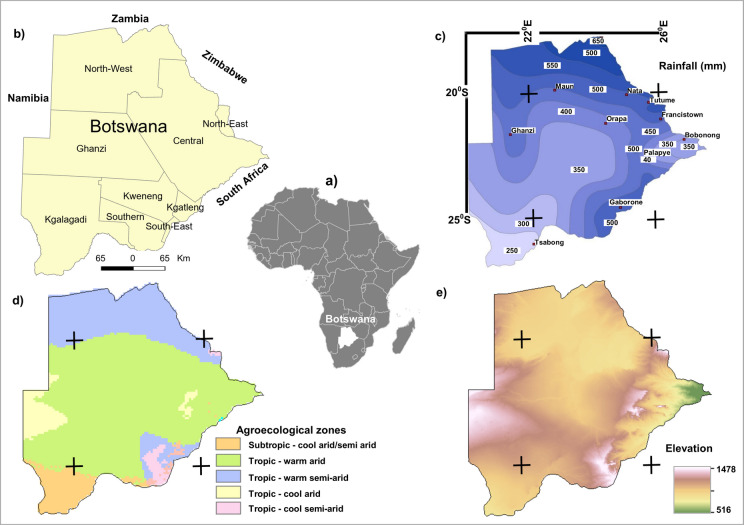
Study Area **a**) Africa insert, **b**) Botswana district administrative map (Akinyemi & Abiodun, 2019), **c**) Rainfall distribution and sites of meteorological stations (Statistics Botswana, 2019b), **d**) Agroecological zones (International Food Policy Research Institute 2015), **e**) Elevation (GTOPO30, USGS 2025)

Botswana is a landlocked country in southern Africa bounded on the West by Namibia, Zambia and Zimbabwe in the north and South Africa to the south (Fig. 1). With an area of ~ 580,000 km^2^, it has a human population of 2,154,863 inhabitants as of 2017 (Statistics Botswana, 2018a). Most of the population is concentrated in the eastern part, where the majority of rainfed agricultural holdings are situated due to a rainfall gradient increasing northwards (Statistics Botswana, 2015b).

### Climate

Botswana has a semi-arid, hot steppe climate — Koppen’s BSh classification and warm desert (arid) climate — Koppen’s BWh (Peel et al., 2007). As a dryland, the natural and human systems in Botswana are highly susceptible to climate change. The country is highly vulnerable to drought since it depends mostly on rainfall for freshwater supply, groundwater recharge and agriculture (Byakatonda et al., 2018b). The annual national average rainfall is below 500 mm as rainfall ranges from 250 mm in the southwest to 650 mm in the northeast (FAO, 2005), while the inter-annual rainfall variability is high (Kenabatho et al., 2012). Occurring mainly in the summer (October to March), intense rains account for most of the rainfall (Tsheko, 2004). The average monthly temperatures range between 15 °C and 27 °C, and the daytime temperature occasionally reaches 40 °C (Nkemelang et al., 2018). The annual evaporation rate is between 1,800–2,100 mm year^−1^, far exceeding rainfall amounts (i.e., 250–650 mm year^−1^) (Byakatonda et al., 2018a).

### Agriculture

Agriculture in Botswana is highly vulnerable to climate change, variability and extremes because of its semi-arid context (Akinyemi & Abiodun, 2019; Mugari et al., 2020). Botswana’s two agricultural production systems for crop production and livestock rearing are the traditional (mostly communal) and commercial systems. The main distinctions between the traditional and commercial systems are the differences in land tenure, agricultural inputs and modern technologies (FAO, 2005). Most farms in Botswana are still under the traditional system, with about 41,043 land holdings over a total area of 204,965 ha as of 2015. In contrast, the commercial system has about 305 land holdings covering 54,691 ha. However, agricultural productivity is higher in the commercial system than in the traditional system. Government programs include the National Agricultural Master Plan for Arable Agriculture and Dairy Development program (Ministry of Agriculture – MOA, 2002, Statistics Botswana 2018a and b). Although the contribution of agriculture to the gross domestic product (GDP) decreased from about 40% in 1966 at independence to less than 2% in 2024 (Q1), agriculture is of great importance to sustaining rural livelihoods (Manthe-Tsuaneng, 2014; Statistics Botswana, 2024). Hence, agriculture remains a priority sector viewed by the government as having the potential to reduce rural poverty.

### Drought planning and management

As drought is a recurring natural hazard in Botswana, there is the risk of drought and its impacts on croplands and grasslands (UNCCD, 2024). The Botswana Statistics office produces a periodic natural hazard digest that features drought impacts on agricultural production (i.e., crop and livestock) (Statistics Botswana, 2019a). Botswana uses a comprehensive approach to drought management that includes agriculture, rangeland management, water resources, tourism and industry. This allows for cross-sectoral interaction with the potential to shift from reactive crisis response to proactive management of drought aimed at building the long-term resilience of social and ecological systems (Batisani, 2020). Several instruments have been developed for drought planning and management (Southern African Drought Initiative —SADRI, 2021). These include the National Drought Plan (NDP) 2020 aiming to integrate early warning systems, vulnerability assessments and coordinated drought response mechanisms across sectors (Batisani, 2020).

The socioeconomic impacts of drought have long shaped government policies of interventions (Vierich & Sheppard, 1980; Holm & Morgan, 1985; Seekings, 2016). To ensure emergency water supply in the event of hydrological drought, three large water supply schemes were recently developed (World Bank, 2024). A survey of drought and household food security is conducted annually to assess vegetation condition and agriculture during the rainy season. The survey forms the basis for the declaration of an official drought year, which necessitates government intervention such as relief to farmers and minimizing suffering for vulnerable groups during a drought (Rural Development Council — RDC, 2019). It can also inform food import planning to mitigate the impact of a deficit in food supply due to a shortfall in production during droughts (RDC, 2017). Initial inputs into the annual drought and household food security survey are remote sensing drought indices such as the vegetation condition index (VCI), rainfall anomaly and decile maps and monthly drought risk maps produced by the agro-meteorological services (Manthe-Tsuaneng, 2014; Ifejika Speranza et al., 2023).

## Data and methods

**Fig. 2:**
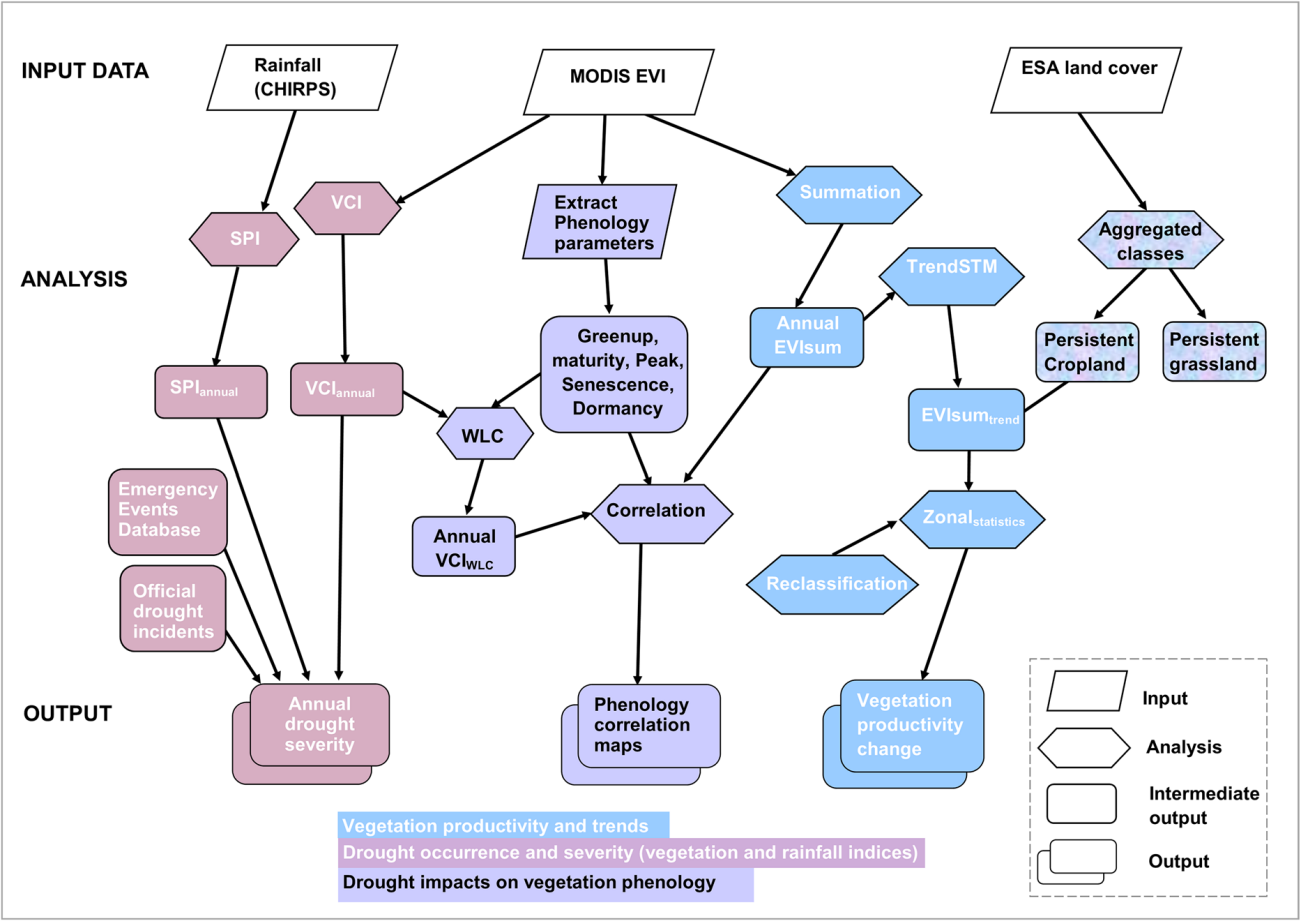
Study workflow used for data processing and analysis. SPI = Standardized Precipitation Index, VCI = Vegetation Condition Index, WLC = Weighted Linear Combination, EVI = Enhanced Vegetation Index, TrendSTM = Season trend model

The datasets and methods employed in this study are described in the methodological flowchart (Fig. 2). All datasets are spatial and were clipped to the Botswana international boundary for consistency before analysis. To fit our objectives, all datasets were compiled on an annual basis using the climatological year from the first of July to the end of June in the subsequent year. This climatological year helps to better capture the entire austral summer raining season between October and March (Maruatona & Moses, 2021).

### Data

#### Vegetation indices

The 16-day Enhanced Vegetation Index (EVI) time-series (250 m) from the Moderate Resolution Imaging Spectroradiometer (MODIS) Terra MOD13Q1.061 (2000–2020) were used in this study. EVI is widely used as a proxy of vegetation greenness as it captures canopy photosynthetic capacity and offers higher radiometric and spatial consistency by selecting the best-quality pixel across the period (Didan et al., 2015). EVI is especially well-suited for semi-arid regions like Botswana because it minimizes soil background noise and atmospheric interference, which are common challenges in sparse vegetation landscapes. Unlike NDVI, which is available in 10-day composites, there is no operational 10-day EVI product, positioning the 16-day EVI as the most robust option for detecting drought stress and monitoring vegetation dynamics under water-limited conditions (Sims et al., 2021). Moreover, studies have found that EVI accounts for factors affecting agricultural conditions such as soil type, radiation or temperature and that the 16-day data better incorporates crop growth and weather information than the monthly products (Cao et al., 2021; Kuri et al., 2019).

#### Vegetation phenology

Vegetation phenology was extracted from the MODIS (MCD12Q2.006) land cover dynamics (MODIS_LCD) data with 500 m spatial resolution (Friedl et al., 2019; Gray et al., 2019). The 250 m EVI data was resampled to match the 500 m phenology data using bilinear resampling as the latter dataset is also EVI-based. As it contains the total number of growing-cycles detected yearly, indicators extracted include vegetation greenup, greenup midpoint, maturity, peak, senescence and dormancy. The greenup — start of the growing-cycle, the greenup midpoint and maturity are measured as the first date when EVI crosses 15%, 50% and 90% of the greenup segment EVI amplitude. Peak greenness is the date EVI reaches the segment maximum. The start of senescence, senescence midpoint and dormancy are the last dates when EVI crosses 90%, 50% and 15% of the greendown segment EVI amplitude.

#### Land cover data

To limit the assessment of drought impacts to agricultural areas, the 300 m European Space Agency (ESA) land cover data (CCI-LC v.2.0.7) was used to derive maps of persistent croplands and persistent grasslands in the absence of a national agricultural mask. The CCI-LC is a consistent annual series of multi‐sensor land cover datasets (ESA, 2017). Annual land cover maps were needed to create the persistent cropland and grassland masks because only pixels that met the condition of remaining either cropland or grassland throughout the 21-year study period (i.e., between 2000 and 2020) were retained for further analysis. Since the CCI-LC provided annual land cover data, it was used to ensure the long-term persistence of cropland and grassland pixels in the masks. Each annual map has more than 30 land cover categories over Botswana, of which six were aggregated into the cropland class and a grassland class (included as supplementary information, see Table S1). For example, the cropland class is comprised mostly of rainfed cropland areas and mosaic natural vegetation types with varying percentages of croplands, whereas the grassland class comprises only grassland.

#### Rainfall data

The monthly rainfall 0.05° (~ 5 km^2^) grid data of the Rainfall Estimates from Rain Gauge and Satellite Observations (CHIRPS) was used to compute the Standardized Precipitation Index (SPI) over Botswana (Funk et al., 2015). Several studies have shown that CHIRPS gives a credible simulation of daily and monthly rainfall in Africa, such as in Mozambique, Nigeria, Uganda, Ethiopia and Zambia (Funk et al., 2015; Maidment et al., 2017; Toté et al., 2015). The SPI was computed to verify the assessed drought conditions based on the vegetation indices.

### Methods

Analysis of vegetation productivity and changes was conducted and related to drought severity and impacts on phenology across Botswana. Analysis was conducted in croplands and grasslands to identify agriculture-specific impacts of drought. To create the persistent cropland and grassland mask for Botswana, we extracted all cropland and grassland pixels separately as binary classes (i.e., presence or absence). Using cropland as an example, first, an annual binary map of cropland and non-cropland pixels was created. Afterwards, the binary maps were overlaid and pixels extracted that met the condition of being a cropland in each year throughout the 21-year study period (i.e., between 2000 and 2020). Based on the identified pixels that remained cropland throughout the time-series, a final map of permanent cropland extent was derived to mask out all other non-cropland pixels. To integrate agriculture beyond croplands in the drought assessment, a map of persistent grassland, such as for livestock keeping, was made for comparison. Only these cropland and grassland pixels were retained for further analysis. Since only the CCI-LC provided annual land cover data covering the desired study period, it was used to create the cropland and grassland masks. This masking procedure to exclude non-grassland and non-cropland pixels from the masks was adopted to minimize the mixed-pixel effects on the analysis results, especially considering that these are mostly fragmented agricultural landscapes.

#### Vegetation productivity and trends

EVI-based indicators derived from the plants’ spectral reflectance were used as proxies for capturing vegetation productivity and changes in the time-series. The sum of EVI (EVI_sum_) was estimated to capture vegetation productivity (Eq. 1). EVI_sum_ is the total annual sum of EVI for each climatological year, i.e. July to June the following year, in the time-series.

1 $$EV{I}_{sum}={\sum }_{t=1}^{n}\left(2.5\frac{\rho ni{r}_{t}-\rho re{d}_{t}}{\left(\rho ni{r}_{t}+6\rho re{d}_{t}-7.5\rho blu{e}_{t}\right)+1}\right)$$

where *t* indexes each time point (16-day), *n* is the number of observations in the year, ρ*blue*_*t*_, ρ*red*_*t*_ and ρ*NIR*_*t*_ are spectral reflectance in the blue, red and near-infrared bands at time t, respectively.

Changes in vegetation productivity were analyzed based on the trend of the EVI_sum_ using the TrendSTM, a season-trend model in R. The significance of the trends found was assessed using the Mann‐Kendall (MK), a non‐parametric test that is robust to outliers (Neeti and Eastman, 2011). MK estimates trends in the time-series by quantifying the rate of change in EVI_sum_ for each pixel. The output (EVIsum_trend_) was used to gauge these changes in vegetation productivity based on the trend coefficient tau (τ). The coefficient ranging between −1 and + 1 signifies a decreasing trend for negative values (indicating a continuing browning) or an increasing trend for positives (indicating a continuing greening), whereas values around 0 are in more stable conditions. This formed the basis for interpreting EVIsum_trend_ as a continuum from decreasing (< 0), stable (0), to increasing (> 0). The significant trends at 95% significance level were afterwards assessed using the GIS zonal statistics function to relate the pixel-level EVIsum_trend_ values to areas of cropland and grassland persistence. The aim was to gauge how long-term changes in vegetation productivity evolved over time in agricultural lands.

## Annual drought severity

Two indices were used in this study for characterizing drought occurrence and severity throughout the time series. These are the remote sensing vegetation-based Vegetation Condition Index (VCI) and the conventional rainfall-based Standardized Precipitation Index (SPI). Using both indices allowed for comparing drought severity with vegetation and rainfall-based methods.

### Weighted vegetation condition index (VCI_wlc_)

The VCI was computed annually from the EVI time-series over 21 years, i.e., between 2000 and 2020. VCI is useful as an agricultural drought indicator as it is suited to capture moisture stress in vegetation due to water deficiency (Akinyemi, 2021). Computed as in Eq. 2, it compares the value of the vegetation index, in this case EVI of a given period* j* (EVI_j_), with the long-term minimum EVI (EVI_ltmin_) and maximum EVI (EVI_lt*max*_).

2 $${VCI}_{j}=\left(\frac{E{VI}_{j}- {EVI}_{ltmin}}{{EVI}_{ltmax}-{EVI}_{ltmin}}\right) x 100$$

The values of VCI range between 0 and 100%, where values close to 0 signify drought-stressed vegetation, while higher values, closer to 100, indicate non-drought conditions (Zambrano et al., 2016). In Botswana, the VCI value of 36% depicts the drought detection point, whereas values below 10% indicate extreme drought conditions (MEWT, 2021).

Since the effects of droughts are most severe at the early stages of plant growth (Graw et al., 2020), the Weighted Linear Combination (WLC) was applied to the VCI to better relate agricultural drought severity to the plants’ growth stages when drought occurred. This entails computing a mean VCI value per seasonal phenological time-block (i.e., greenup, maturity, peak, etc.), which is applied as a weighting to the initial VCI value to derive a final, weighted VCI (VCI_wlc_) for the entire growing season (Graw et al., 2020). VCI_wlc_ was estimated to aid in detecting anomalies in vegetation productivity, which is afterwards used to further confirm the detection of drought and non-drought years in Botswana. Unlike studies differentiating drought severity into classes, the VCI values were not grouped into classes when applying the WLC to allow using the range of values in further analysis. In contrast, the SPI is based only on rainfall data. Widely used to characterize drought in different world regions (Mckee et al., 1993), it is a multiscale drought index applicable from 1 to 24 months, depending on the drought under consideration. This study uses the SPI-3 (i.e., 3-month scale), which is relevant for assessing drought impacts on cropping and herbaceous systems, whereas intermediate periods (e.g., SPI-9 to SPI-12) are more amenable for use when considering perennial systems such as in forestry or agroforestry (Contreras & Hunink, 2015; Garcia-Leon et al., 2019). SPI was computed as in Eq. 3 using the monthly CHIRPS rainfall time-series data for the study period from 2000 to 2020.

3 $$SPI= \frac{\left({Rs}_{i}-{Rs}_{lt}\right)}{\sigma }$$

where *RS*_*i*_ is the observed precipitation, *RS*_*lt*_ is the long-term mean precipitation. SPI classes range from negative values, signifying various drought severities, to positive values, signifying wetness, with values close to 0 indicating near-normal or normal conditions.

The evolution of vegetation phenology indicators was assessed to further gauge the impacts of drought during the growing cycle. We sought shifts in agriculture-related phenology as driven by droughts in the time series. The analysis conducted includes extracting the MODIS-based phenology indicators (i.e., greenup, maturity, peak, etc.) for each annual growing cycle between 2000 and 2020 to assess the dynamics and patterns of the phenometrics in agricultural land. Second, we correlated these indicators with the annual EVI_sum_ (i.e., vegetation productivity) and VCI_wlc_ (the weighted VCI drought severity index) using the Pearson correlation. The aim is to identify how the development of these phenology indicators in agricultural lands, specifically in grasslands and croplands, is related to drought conditions.

## Results

The summation of EVI (EVI_sum_) was the first step in assessing how vegetation productivity and phenology changed over the two-decade study period.

### Vegetation productivity

**Fig. 3:**
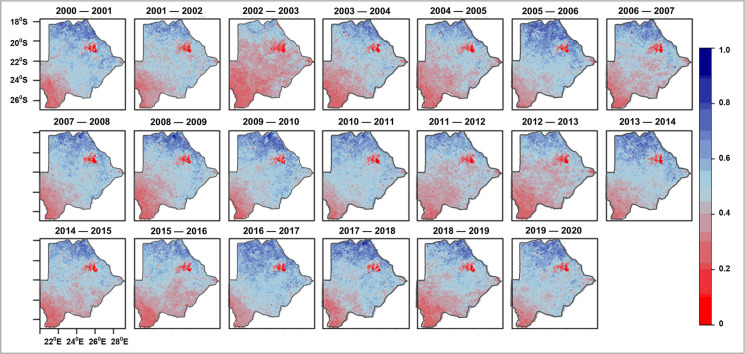
Sum of EVI as an indicator of vegetation productivity over Botswana (2000–2020)

The annual EVI_sum_ map for each year shows how vegetation productivity varied in space and time in Botswana (Fig. 3). Very low vegetation cover was evident over almost the entire country in years 2002–2005, 2006–2007, 2011–2013, 2014–2016 and 2018–2019, which coincide with drought years. Providing insights about total vegetation productivity, the EVI_sum_ depicts the southwest as consistently low compared to the north, with consistently more vegetation cover.

### Vegetation productivity change over agricultural lands

**Fig. 4:**
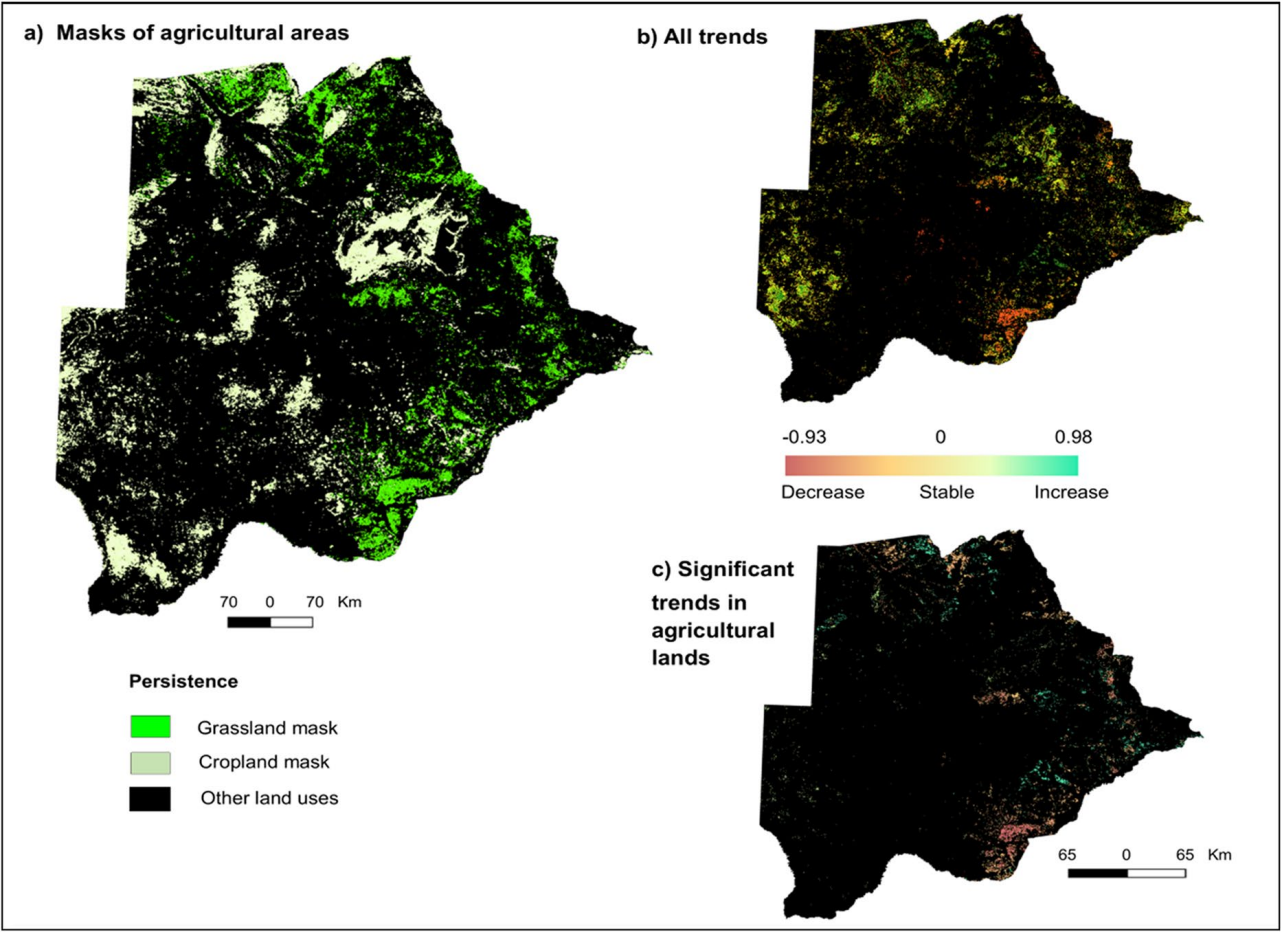
**a** Spatial extent of persistent croplands and grasslands, **b** All vegetation productivity trends, **c** Significant trends in agricultural land areas at 95% confidence level

To infer drought impacts on agricultural productivity, we examined how changes in vegetation productivity (i.e., EVIsum_trend_), varied across persistent grasslands and croplands. Areas of persistent croplands and grasslands amounted to about 2.24 million ha (~ 3.8%) and 2.8 million ha (~ 4.8%) of Botswana’s land area, respectively (Fig. 4a).

Persistent grasslands occurred mostly in the western half from north to south, whereas persistent croplands were mostly to the southeast, eastern and northern parts of the country (Fig. 4a). Together, they form persistent agricultural land areas over which we mapped tau trend coefficients (Fig. 4b). Decreasing vegetation productivity (tau < 0), characterized by negative vegetation trends, affected about 604,800 ha (~ 27%) of croplands mostly in southeast Botswana and about 110,000 ha (3.9%) of grasslands. Areas with stable vegetation productivity (tau = 0) amounted to about a million ha (~ 46%) in croplands and about 31,000 ha (1%) in grasslands. Vegetation productivity increased (tau > 0) in about 600,000 ha (~ 26%) of croplands and 2.6 million ha (95%) of grasslands, although the increasing trends found in grasslands were mostly not statistically significant.

### Drought occurrence and severity from vegetation and rainfall indices

**Fig. 5:**
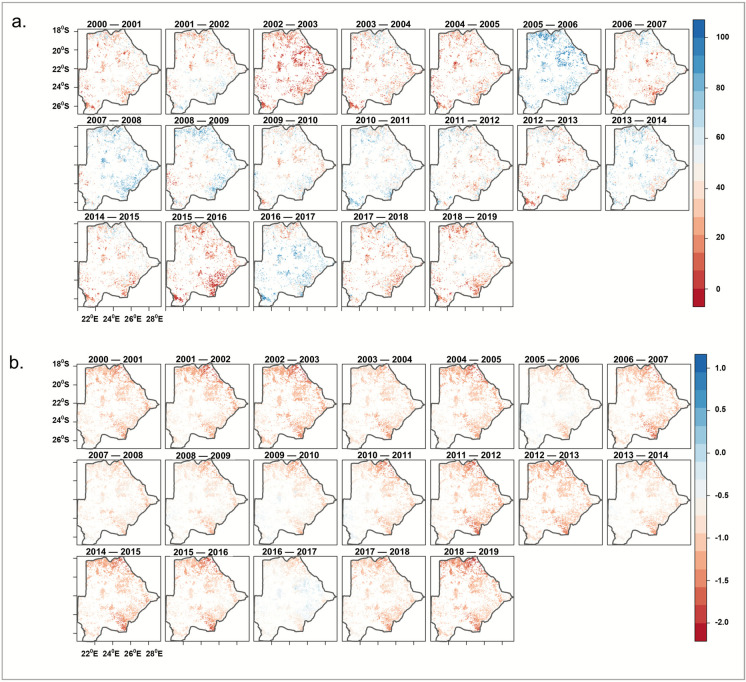
Annual agricultural drought severity over agricultural lands in Botswana, **a** Weighted Vegetation Condition Index (VCI_wlc_), **b** Standardized Precipitation Index (SPI)

The severity of agricultural drought on vegetation productivity in croplands and grasslands (2000–2020) was assessed based on two drought indices. Agricultural drought was computed with the vegetation-based VCI_wlc_ and the precipitation-based SPI, as shown in Fig. 5a and b. From the VCI_wlc_, the most drought-stricken years, when most of the country was affected, were 2000–2005, 2006–2007, 2011–2013, 2014–2016 and 2017–2019. The VCI_wlc_ drought patterns during these periods largely match those of the SPI. Comparing the outputs from vegetation and rainfall drought indices in persistent agricultural lands allows to infer how drought impacted vegetation productivity in relation to the annual rainfall regime.

**Fig. 6:**
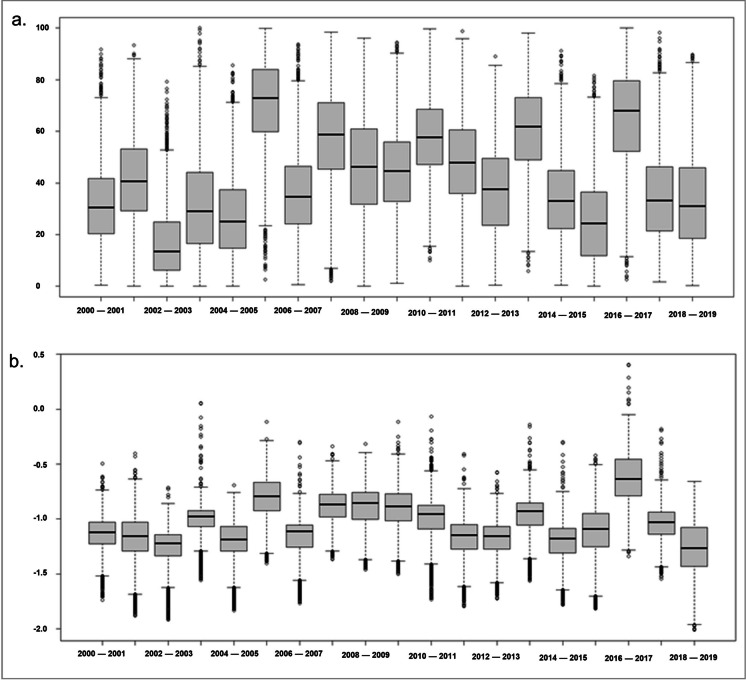
Drought severity across persistent croplands and grasslands in Botswana (2000–2020) based on **a** vegetation index (VCI_wlc_),** b** rainfall index (SPI). Each boxplot shows the spread of the simulations (minimum, first quartile, median, third quartile, and maximum)

The map depictions in Fig. 5 show different drought severity patterns across the country, particularly over agricultural lands. Further confirming the identified drought years (Fig. 6), the boxplots (Fig. 6a) revealed that 2002–2003 was the year with the most severe drought. In contrast, 2005–2006 and 2016–2017 were the years with the least drought impacts on vegetation productivity as evident with the high VCI_wlc_. Similar patterns can be seen in the boxplot analysis for SPI (Fig. 6b).

**Fig. 7:**
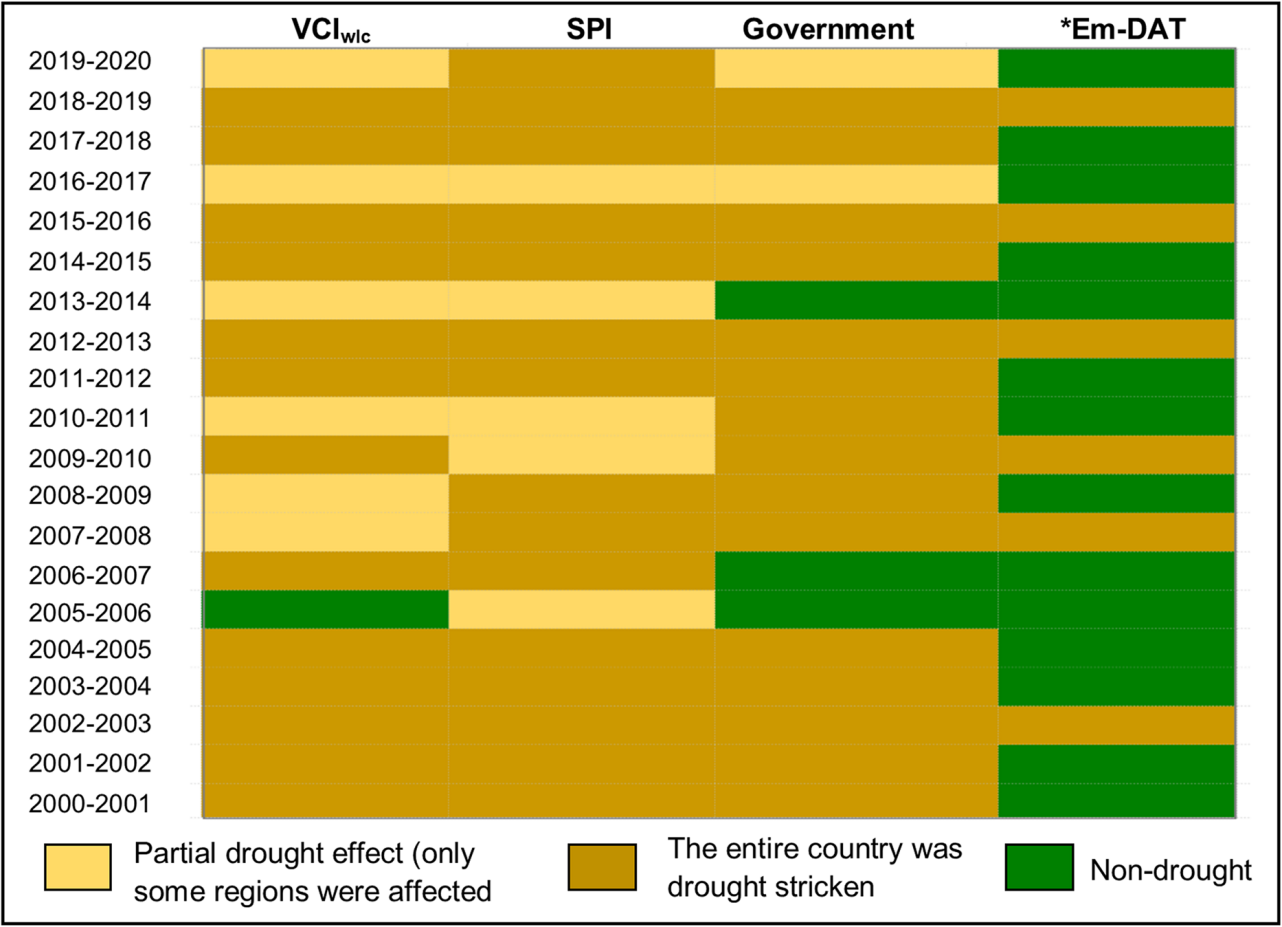
Drought occurrence by indices and sources. *Data is over Southern Africa, including Botswana

Further confirmation of drought occurrences identified from the vegetation and rainfall-based indices was sought. We used the official record of government-declared drought years and the Emergency Events Database (EM-DAT, 2025) (Fig. 7). The years declared officially as drought stricken by the government within our study period are 2001–2005, 2007–2008, 2008–2009, 2009–2013, 2014–2016, 2017–2019, whereas in 2016–2017 and 2019–2020, drought declaration was partial to cover only the few affected regions (Statistics Botswana, 2018b; Akinyemi, 2021). In the past, drought occurrences were only recorded for certain areas such as the former Northeast and Central districts (1961–1965), Bobirwa (1970–1980) and the entire country (1981–1987) (Statistics Botswana 2018a, b, c). The identified drought years from both the vegetation and rainfall-based indices were similar to the government-declared drought years than to the drought incidents recorded in the EM-DAT. For example, 2011–2012 and 2014–2015 were officially declared drought years, but these years were not recorded in EM-DAT.

### Relating vegetation phenology to drought severity impacts

**Fig. 8:**
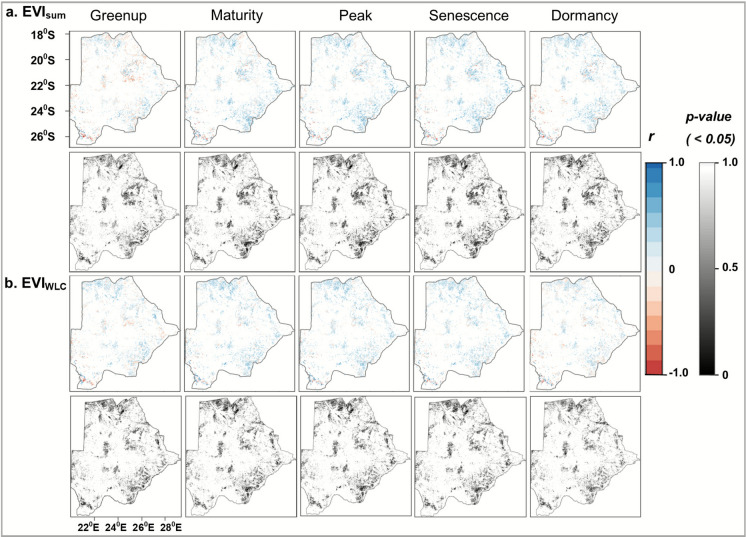
Correlation of the vegetation phenology indicators during 2000 to 2020 **a** Correlating EVI_sum_, indicating vegetation productivity to phenology and **b** Correlating VCI_wlc_, indicating the weighted agricultural drought severity to phenology. *r* — represents the Pearson correlation coefficients

We examined how the five phenology indicators (i.e., vegetation greenup, maturity, peak, senescence and dormancy) are correlated to the annual EVI_sum_ (a proxy of vegetation productivity) and VCI_wlc_ (the weighted agricultural drought severity index) in persistent agricultural lands (Fig. 8). The Figure depicts how these agriculture-related phenometrics are interlinked with drought conditions. For example, we found that late greenup — the start of season — correlates with lower values of EVI_sum_ signifying declining vegetation productivity and lower VCI_wlc_ values signifying higher agricultural drought severity. There is mostly a strong, positive relationship between EVI_sum_ and the phenometrics, especially in the north and southeast, whereas the relationship is strong but negative in the central and southwestern parts of Botswana. These patterns of the relationships with EVI_sum_ are similar to those with the VCI_wlc_. This is intuitive as drought effects captured in the VCI_wlc_ is reflected in the observed changes to vegetation productivity in the EVI_sum_.

**Fig. 9:**
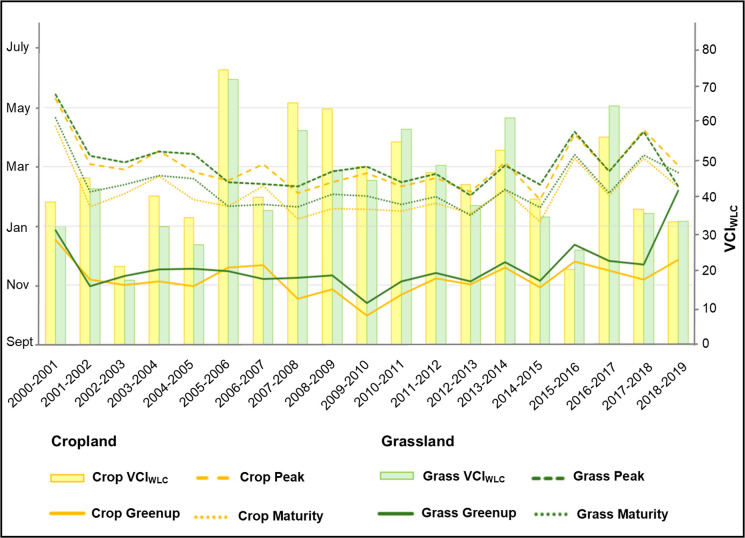
Agriculture-related drought severity and phenology in persistent croplands and grasslands in Botswana

We further examined how these five phenology indicators evolved in the VCI_wlc_ time-series. To better detect whether there are drought-induced phenological shifts over agricultural lands, this necessitated differentiating between croplands and grasslands with regard to drought severity by months during the different growth stages such as greenup or peak (Fig. 9).

Based on the VCI_wlc_, grasslands were more affected by drought as this class consistently had lower VCI_wlc_ values than croplands except for five years in the time-series. Regarding phenology, the start of the season (i.e., greenup) often occurred later in grasslands than in croplands. Especially during drought, grasslands show a later start in greenup than croplands while the peak period often overlaps with croplands. The shift in vegetation phenology in Botswana throughout the entire time-series was about 58 days delay in greenup between the earliest and latest detection, whereas it was between 60 and 61 days for peak, senescence and dormancy.

## Discussion

### Vegetation productivity and change in persistent agricultural lands

Our definition of persistent croplands and grasslands implied long-term permanence, involving the removal of all cropland and grassland pixels engaged in land use change between 2000 and 2020 from the analysis. Detecting changes to vegetation productivity was based on trend analysis (i.e., decreasing, stable—normal conditions and increasing trends), which varied temporally and by land use. For example, vegetation productivity was stable in about half of the persistent croplands compared to only 1% of the grasslands. This implies that vegetation productivity did not change much in the majority of croplands as compared to grasslands. However, vegetation productivity increased in the majority of grasslands compared to only about a third of croplands, and productivity declined more in croplands than in grasslands. These findings provide empirical evidence that ecosystem changes can occur within the same class in the absence of land use changes (Fu et al., 2015; Muro et al., 2016). Relating this to agricultural drought impacts, vegetation productivity in grasslands was consistently lower than in croplands, especially during droughts. Our findings agree with previous studies that found that rangeland’s biomass reduced by about 30% (Setshwaelo, 2000) and cropland’s biomass reduced by about 36% (Akinyemi et al., 2021) in Botswana. The values of changes in vegetation productivity in cropland and grassland could not be directly compared to Kombani et al. (2025) as their results were not disaggregated by land use.

Regarding changes to vegetation productivity, we found negative vegetation trends in the southeast of Botswana, especially in croplands and increasing trends in the west over Ghanzi and southwest Kgalagadi. These findings are largely in line with Akinyemi et al. (2021), who found declining trends in productivity mostly in the southeast and stable but stressed productivity in the southwest based on MODIS 250 m NDVI data between 2000 and 2015. The declining trends can be partly explained by the high interannual variability in vegetation and rainfall conditions, especially in the southeast of Botswana (Mberego, 2016). Some studies have attributed the increasing trends in vegetation productivity in the majority of grasslands, especially in the southwest, to bush encroachment in the rangelands (e.g., Akinyemi & Kgomo, 2019; Reed et al., 2015). Bush encroachment may be detrimental in cattle-based systems if it fosters the reduction in tree species diversity, increased woodiness and unpalatable grass species (Thomas et al., 2012).

### Identified drought and non-drought years

Results from the vegetation-based agricultural drought (VCI_wlc_) and rainfall-based drought indices reveal drought years in which the entire country or parts were affected. There was no year when all of Botswana was completely drought-free. In other words, drought-stricken areas were found in parts of Botswana in any given year. For example, when much of Botswana experienced no droughts, such as in 2005–2006, 2007–2008 and 2016–2017, some parts of the southwest and southeast were severely affected.

We compared the identified droughts with both government declarations and the EM-DAT. The discrepancy with the latter might be due to missing data and the fact that the database also considers the number of people affected by drought (Jones et al., 2023). The discrepancies in the identified drought years also show that the definitions and the thresholds used to detect drought and non-drought occurrences play a crucial role in drought assessment as input to disaster risk management. Thresholds to be used at local or national levels for decision making ought to be streamlined in comparison to the regional or global levels (Harm et al., 2022; Nohrstedt et al., 2022).

### Agricultural drought impacts regarding phenology

We assessed the patterns of phenology indicators and correlated them with the EVI_sum_ and VCI_wlc_. To specifically capture agriculture-related phenology, we examined how vegetation phenology evolved over time in persistent croplands and grasslands. For example, we found later greenup, mostly in grasslands signifying the delayed start of the season. This correlated highly with reduced vegetation productivity and severe agricultural droughts as evidenced by lower EVI_sum_ and VCI_wlc_ values, respectively, mostly during droughts. Such delayed onset of greenup was more pronounced in grasslands than in croplands, indicating a shortened duration of vegetation greenup. Despite the increasing trends found in the majority of grasslands, vegetation productivity was generally lower in grasslands than in croplands. The lesser effects of droughts on cropland compared to grassland might be partly explained by irrigation schemes used for crop production and the increasing adoption of drought-resistant crops. Moreover, previous studies have noted that rangelands are often degraded due to overgrazing, especially in communal lands, which can exacerbate the impacts of drought on grasslands (e.g., Akinyemi & Kgomo, 2019; Akinyemi et al., 2021). Thus, these changing phenological patterns in the growth stages in the series provide evidence that vegetation phenology is highly dynamic and responsive to drought conditions in drylands.

The sequence and interconnectedness of vegetation phenology stages suggest that drought-induced changes in one stage may also impact subsequent stages, necessitating further research on the legacy effects of drought on phenology, as these might reflect on the productivity outcomes (e.g., crop yield). Vegetation phenology studies elsewhere have found some changes associated with phenology stages. As early as 2007, Rivero et al., (2007) found an association between drought and accelerated leaf senescence and leaf abscission in perennials, which serves as a means for plants to decrease canopy size as a means for plant survival in a bid to quickly complete the growth cycle under drought stress. Wang et al. (2016) also noted that between 1982–2015, changes in spring greenup onset influenced the timing and amplitude of the growth peak in northeast China. The change patterns in vegetation phenology in croplands and corroborated in grasslands over Botswana provide valuable insights into how vegetation responds over time to droughts. Although beyond the scope of this study, the attribution of the observed change patterns needs further examination as other environmental and human factors may have confounding effects besides drought (Haile et al., 2019). Thus, this study highlights the importance of understanding and monitoring vegetation phenology linked to drought conditions in dryland contexts such as Botswana.

## Concluding remarks

We estimated vegetation productivity and examined how it evolved annually using Remote Sensing time-series data within a two-decade study period (2000–2020). The study identified drought and non-drought years and compared the drought severity obtained from the weighted Remote Sensing vegetation-based agricultural drought index (VCI_wlc_) with that of the conventional, rainfall-based SPI. While rainfall data is often only available in sparse station locations across the country, the additional use of Remote Sensing-based vegetation indices, disaggregated by land use or agroecological zones, is complementary.

That droughts were experienced across much of Botswana during most of the years in the series reveals Botswana’s vulnerability to drought. The study examined the patterns and dynamics of five phenology indicators (i.e., greenup, maturity, peak, senescence and dormancy). It then examined how droughts have impacted vegetation productivity and phenology over two decades. Correlating these vegetation variables with agricultural drought severity, we better understood the changes in vegetation productivity and phenology in response to droughts. We found differences in the severity and impacts of droughts on agricultural lands, which made for a comparison between croplands and grasslands. Considering the importance of grasslands for livestock keeping in Botswana, the latter represents a valuable natural resource for alternative coping strategies, as rainfed crop production often fails during droughts. Several change patterns in vegetation productivity and phenology were found. Notable patterns include that, grasslands consistently have lower vegetation productivity compared to croplands. Such lower vegetation productivity, coupled with a delayed and highly variable vegetation greenup, which was more pronounced in grasslands, can result in inconsistent forage availability for livestock. These can negatively impact livestock health and productivity, which is critical for Botswana’s economy as a beef exporter.

Considering Botswana’s differing climatic and agricultural conditions, our results can be further clarified by disaggregating into agroecological regions. This is a study limitation which future studies can explore. The annual 300 m ESA land cover data used in this study fulfilled the requirement for creating the permanent grassland and cropland masks to ensure long-term persistence and homogeneity of pixels. Moreover, the 300 m resolution of the ESA data is closer to the 250 m MODIS EVI data. Our methods can work with improved, higher-resolution image datasets such as the 10 m Sentinel-2. However, Sentinel-2 time-series data archive is still too short for trend analysis, as 16 years of data would have been missed if Sentinel-2 was used in this study. As remote sensing-based estimates are from indicators, field-based data are essential to further calibrate and validate the accuracy to minimize misclassification or errors in phenology estimates. National-level assessments, such as those conducted in this study for Botswana, require balancing data availability and resolution with the computational demands of the large area analysis.

The good level of agreement on drought occurrences between the vegetation-based agricultural drought indices and rainfall-based SPI and published sources (e.g., government official declaration) attests to the credibility of Remote Sensing image time-series data for simulating drought and its impacts in drylands. Considering the coupling of rainfall and vegetation, it is essential and even more critical for Botswana to monitor both rainfall and vegetation changes for drought planning and management.

## Supplementary Information

Below is the link to the electronic supplementary material.

ESM 1 (PDF 342 KB)

## Data Availability

The Enhanced Vegetation Index data (MOD13Q1.061) and Phenology data (MCD12Q2.006) are available from the Moderate Resolution Imaging Spectroradiometer (MODIS) at [10.5067/MODIS/MOD13Q1.061] (10.5067/MODIS/MOD13Q1.061) and 10.5067/MODIS/MCD12Q2.006 respectively. European Space Agency (ESA) time-series land cover data v.2.0.7 is available at [https://maps.elie.ucl.ac.be/CCI/viewer/download.php] (https://maps.elie.ucl.ac.be/CCI/viewer/download.php) and CHIRPS [https://www.chc.ucsb.edu/data/chirps](https://www.chc.ucsb.edu/data/chirpsE) . EM-DAT (2022) data is available in the Emergency Events Database - Universite catholique de Louvain (UCL) - CRED, D. Guha-Sapir, Brussels, Belgium. https://www.emdat.be/.
