# Supplementary material for: Two decades of agricultural drought impacts: remote sensing insights into vegetation productivity and phenological change in semi-arid Botswana
Source: Environ Monit Assess. 2026 Jan 30;198(2):188. doi: 10.1007/s10661-026-14996-w (PMC12858528; doi:10.1007/s10661-026-14996-w)
Supplement: Supplementary file 1 — (PDF 342 KB) [file 10661_2026_14996_MOESM1_ESM.pdf]

Supplementary Information to Remote Sensing-based assessment of agricultural drought reveals impacts on vegetation productivity and shifts in agricultural phenology in semi-arid Botswana (2000 – 2020)

Felicia O. Akinyemi, Valerie Graw

As a semi-arid context, agriculture in Botswana is highly precarious due to its vulnerability to climate vagaries. With more evidence of the impacts of a changing and warming climate in Botswana such as changing rainy season characteristics, this study examined agricultural drought severity and effects on vegetation productivity and phenology (phenometrics).

1.0 The cropping calendar

The cropping calendar for major crops grown in Botswana is depicted as Fig. S1.

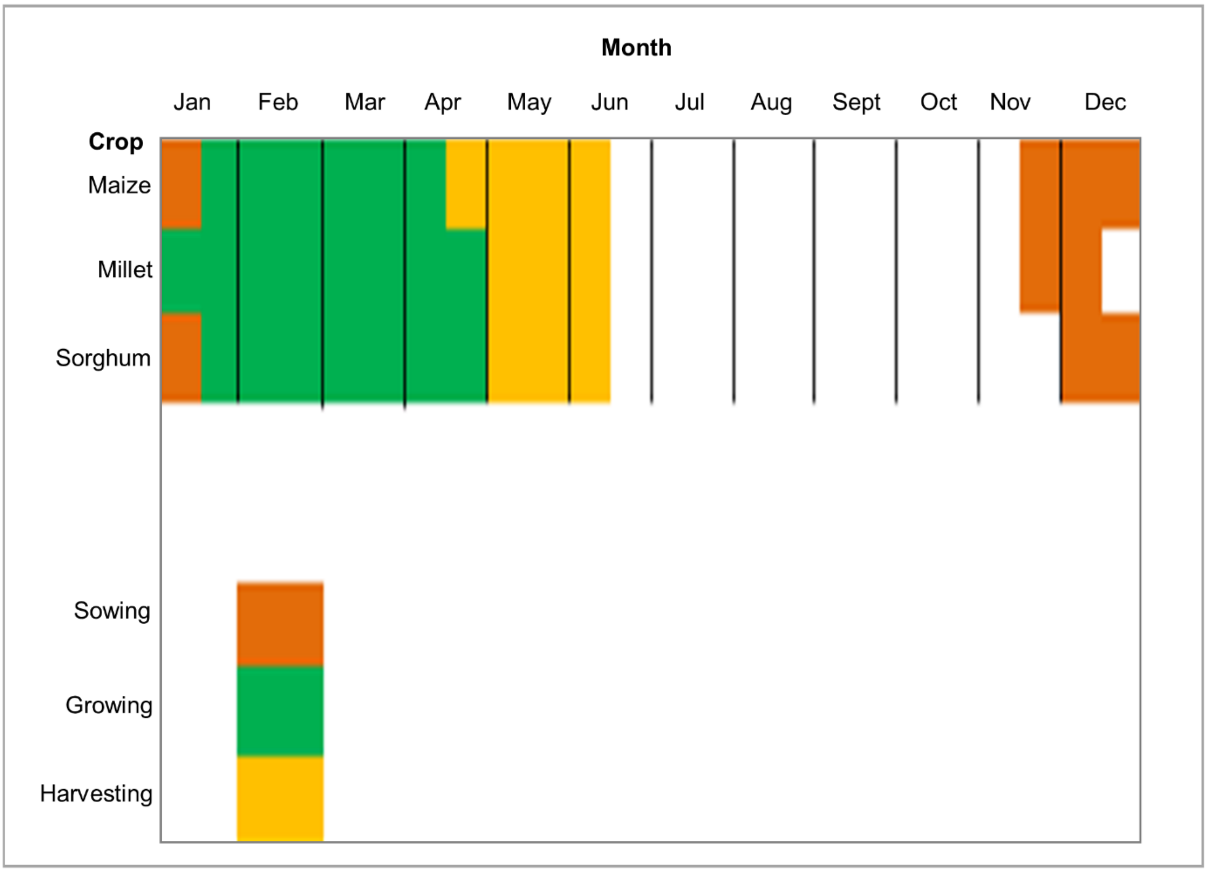

Fig. S1 Cropping calendar for the major crops (FAO, 2016)

2.0 Persistent cropland and grassland

The extent of persistent cropland and grassland were needed to ascertain drought severity on agricultural lands. Datasets of cropland and grassland extent were derived from the European Space Agency (ESA) Climate Change Initiative land cover data (CCI-LC v.2.0.7). ESA CCI-LC land cover data has about 35 land cover classes (ESA, 2017). The aggregated classes are described in Table S1.

Table S1: Cropland and grassland classes aggregated for persistence in the study

Code	CCI-LC classes	New classes
10	Cropland, rainfed	Cropland
11	Herbaceous cover	
12	Tree or shrub cover	
20	Cropland, irrigated or post-flooding	
30	Mosaic cropland (>50%) / natural vegetation (tree, shrub, herbaceous cover) (<50%)	
40	Mosaic natural vegetation (tree, shrub, herbaceous cover) (>50%) / cropland (<50%)	
130	Grassland	Grassland

3.0 Drought severity hotspots

To detect drought hotspots in Botswana, two drought severity thresholds, the VCI_{wlc} below 36 (VCI_{wlc36}) and below 10 (VCI_{wlc10}) were applied (Fig. S2). Most affected regions under VCI_{wlc36} were the southwest, areas around the eastern tip and the central part of Botswana (Fig. S2ai and ii). Based on this drought severity threshold, about 8% of Botswana experienced between 12 to 19 drought-stricken years in the 21-year period, whereas ~50% experienced between 6 to 8 drought-stricken years. Less affected areas (~28%) were mostly in the north and central parts, with five or less occurrences of drought years. Using the stricter VCI_{wlc10} threshold, only 9% of Botswana experienced 3 to 5 of this very extreme drought conditions in the southwest such as the Kgalagadi District and southern parts of Ghanzi District. In contrast, 19% experienced this very extreme drought-stricken years twice and 52% experienced it only once (Fig. S2bi and ii).

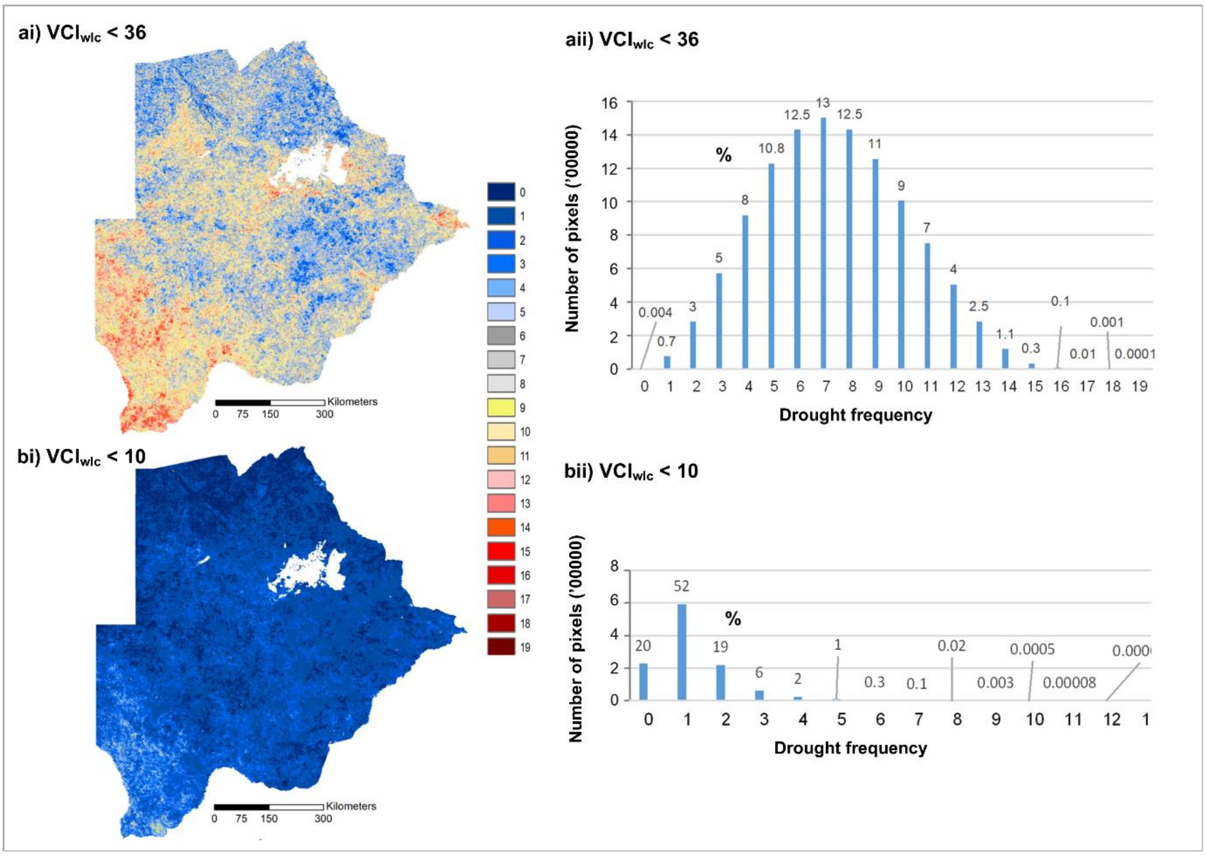

Fig. S2. Heatmaps of drought severity for two thresholds of weighted VCI (VCI_{wlc36} and VCI_{wlc10}), ai) The less than 36 weighted VCI threshold is for values between 0 and 35 signifying extreme drought conditions in Botswana, bi) The less than 10 weighted VCI threshold is for values between 0 and 9 signifying very extreme drought, aii) Drought frequencies (i.e., the occurrences of drought) for the less than 36 weighted VCI threshold, bii) Drought frequencies for the less than 10 weighted VCI threshold. The percentages in the graph represent the proportion of land area experiencing particular numbers of drought occurrences during the 21-year of study.

References

European Space Agency (ESA) (2017). Legend of the global CCI-LC maps, based on LCCS. Accessed 23 March 2025 https://maps.elie.ucl.ac.be/CCI/viewer/download/CCI-LC_Maps_Legend.pdf

ESA (2022). Climate Change Initiative land cover data (CCI-LC v.2.0.7, https://www.esa-landcover-cci.org/).

FAO (2016). Botswana: GIEWS country brief. Accessed 23 March 2025 https://reliefweb.int/sites/reliefweb.int/files/resources/BWA_3.pdf
